# Retraction: a case of meningitis due to *Achromobacter xylosoxidans* in a child with a polymalformative syndrome: a case report

**DOI:** 10.11604/pamj.2021.40.89.31949

**Published:** 2021-10-12

**Authors:** The Pan African Medical Journal

**Affiliations:** 1 Park Suite Building, Parkland Road, Nairobi, Kenya

**Keywords:** Retraction, case report, PAMJ

## Abstract

This article retracts the publication “Achromobacter xylosoxidans in a child with a polymalformative syndrome: a case report” by Mehdi Borni et al. for instances of image manipulation and plagiarism.

## Retraction

It was brought to our attention that the article *“A case of meningitis due to Achromobacter xylosoxidans in a child with a polymalformative syndrome: a case report”* by Mehdi Borni, Mohammed Znazen, Fatma Chaker Borni, Mohamed Zaher Boudawara published in the Pan African Medical Journal [1] contains evidence of image theft and manipulation.

Our internal investigation concluded that this article has clear evidence of image theft, image manipulation and plagiarism.

First, figure 3 Panel A is similar to figure 1 in the article *“Pulmonary Infection Caused by Achromobacter xylosoxidans in a Patient with Carcinoma of Epiglottis: A Rare Case”* [2]. It appears that a portion of Figure 1 in the above-mentioned article was copied, and manipulated.

Secondly, figure 3 Panel B is similar to Figure 4 in the article *“Nosocomial Achromobacter xylosoxidans Infection Presenting as a Cavitary Lung Lesion in a Lung Cancer Patient”* [3]. The image was reused after a slight color modification.

Our investigation additionally uncovered other expression of concerns of image manipulation and theft in articles published by author Mehdi Borni [4,5].

The authors could not provide a reasonable explanation for our findings and do not agree with the retraction. The PAMJ regrets that these grave violations were not identified before publication and sincerely apologizes to the scientific community.
